# Prognostic Factors for Esophageal Squamous Cell Carcinoma—A Population-Based Study in Golestan Province, Iran, a High Incidence Area

**DOI:** 10.1371/journal.pone.0022152

**Published:** 2011-07-21

**Authors:** Karim Aghcheli, Haji-Amin Marjani, Dariush Nasrollahzadeh, Farhad Islami, Ramin Shakeri, Masoud Sotoudeh, Behnoush Abedi-Ardekani, Mohammad-Reza Ghavamnasiri, Ezzatollah Razaei, Elias Khalilipour, Samira Mohtashami, Yasha Makhdoomi, Rabea Rajabzadeh, Shahin Merat, Rasoul Sotoudehmanesh, Shahryar Semnani, Reza Malekzadeh

**Affiliations:** 1 Digestive Disease Research Center, Shariati Hospital, Tehran University of Medical Sciences, Tehran, Iran; 2 Department of Medical Epidemiology and Biostatistics, Karolinska Institute, Stockholm, Sweden; 3 International Agency for Research on Cancer, Lyon, France; 4 Department of Oncology, Omid Hospital, Mashhad University of Medical Sciences, Mashhad, Iran; 5 Golestan Research Center of Gastroenterology and Hepatology, Golestan University of Medical Sciences, Gorgan, Iran; Ohio State University Medical Center, United States of America

## Abstract

Golestan Province in northern Iran is an area with a high incidence of esophageal squamous cell carcinoma (ESCC). We aimed to investigate prognostic factors for ESCC and survival of cases in Golestan, on which little data were available. We followed-up 426 ESCC cases participating in a population-based case-control study. Data were analyzed using the Kaplan–Meier method and the Cox proportional hazard models. Median survival was 7 months. Age at diagnosis was inversely associated with survival, but the association was disappeared with adjustment for treatment. Residing in urban areas (hazard ratio, HR = 0.70; 95% CI 0.54–0.90) and being of non-Turkmen ethnic groups (HR = 0.76; 95% CI 0.61–0.96) were associated with better prognosis. In contrast to other types of tobacco use, nass (a smokeless tobacco product) chewing was associated with a slightly poorer prognosis even in models adjusted for other factors including stage of disease and treatment (HR = 1.38; 95% CI 0.99–1.92). Opium use was associated with poorer prognosis in crude analyses but not in adjusted models. Almost all of potentially curative treatments were associated with longer survival. Prognosis of ESCC in Golestan is very poor. Easier access to treatment facilities may improve the prognosis of ESCC in Golestan. The observed association between nass chewing and poorer prognosis needs further investigations; this association may suggest a possible role for ingestion of nass constituents in prognosis of ESCC.

## Introduction

Esophageal cancer is the 6^th^ cause of cancer death worldwide [1], [2]. The incidence of esophageal cancer has high geographical variation, ranging from 3 per 100,000 person-years in low incidence areas to over 100 per 100,000 in high incidence regions of China and in Golestan Province in northern Iran [3]. Although during the past few decades the number of esophageal adenocarcinoma cases has been increasing in several Western countries [4], [5], ESCC is still the most common histological subtype of esophageal cancer worldwide, particularly in high incidence areas. For example, approximately 90% of all esophageal cancers in Golestan Province are ESCC [6].

Overall, ESCC has a poor prognosis [2], [7]. The outcome of ESCC is mainly determined by age, the stage of cancer at presentation, and type of treatment [8]. Long term survival is mainly reported in a fairly small proportion of cases whose disease is limited to mucosa or submucosa of the oesophagus and who receive appropriate treatment [9], [10]. Several other prognostic factors for ESCC, e.g. sex, race and socioeconomic status [11], [12], have been reported but the results are not as consistent as for the above factors. A few studies have suggested that the prognosis may be influenced by etiologic factors of ESCC, e.g. tobacco use [13], [14], [15]; however, the evidence is limited.

Although ESCC is the most common cancer in Golestan [16], no population-based data from the region are available on prognosis and survival of this cancer . In order to address this issue, we followed up the ESCC cases who had participated in Golestan Case-Control Study, a recent population-based study on upper gastrointestinal cancers in Golestan. We investigated the relation of survival in ESCC cases with several suggested prognostic factors in the literature and risk factors for ESCC in Golestan, including opium and tobacco use [17], [18].

## Materials and Methods

Details of the original case-control study are available elsewhere [6], [18]. Briefly, physicians in the study field were asked to refer all patients suspected of having upper gastrointestinal tract cancer to Atrak Clinic, the only specialized clinic for upper gastrointestinal cancers in Eastern Golestan, where all the patients underwent oesophago-gastro-duodenal videoendoscopy. The process of identifying cases was not based on a screening program; the individuals underwent usual diagnostic procedures. Biopsy specimens were collected using a standard protocol and examined by experienced pathologists. All individuals who diagnosed with ESCC from 2002 to 2007 and agreed to participate were recruited in the current study. Other eligibility criteria were being at least 18 years of age, residing in the study area at the time of registration, and having no history of any other cancer. Results of the Golestan Cancer Registry show that approximately 70% of all incident cases in the study area were referred to Atrak Clinic during the period of study (unpublished results). For almost all cases, ESCC was diagnosed for the first time. Participants were interviewed by trained interviewers who collected detailed information on demographic and lifestyle characteristics, including tobacco, opium and alcohol use, using a structured questionnaire. Data on common tobacco products in the region were collected separately: i.e. cigarette, hookah (water pipe), and nass (a chewing tobacco product which is a mixture of tobacco, lime, and ash). The questionnaire for tobacco and opium use had been validated against urine cotinine, codeine, and morphine level [19]. Location of tumors within the oesophagus was determined by experienced gastroenterologists using data collected during the first endoscopy at Atrak Clinic.

Every three month an experienced nurse contacted the study participants by telephone and asked about their general condition, ability to swallow, and treatment process. In case of death, information on the exact date of death, place of death (hospital versus home), and the reason for death was collected from close relatives of cases. When any telephone number was not available or phone contacts were unsuccessful, a team consisted of a physician and a nurse visited cases or their family at home to collect the above information. The patients were followed from the date of diagnosis until death or the end of the study (June 2008) whichever occurred first.

We attempted to collect reports of laboratory tests, imaging procedures, surgical operative notes, and surgical pathology reports for any participant who underwent those procedures [20]. The planned staging procedure was as following: chest X-ray and abdominal sonography followed by thoracic and abdominal computed tomography (CT) scan (spiral high speed). When all of the above examinations did not show any evidence of metastasis, the cases were referred to Digestive Disease Research Center (DDRC) of Tehran University of Medical Sciences in Tehran for endoscopic ultrasonography of esophageal tumor, which was free of charge. PET-CT scan was not available for staging in the study area or at DDRC during the study period. CT scan was not also available by 2004 in Eastern Golestan. All participants, including those who refused the above staging process, were finally referred to treatment centers. However, a substantial number of cases refused further workup for staging or treatment outside the study field. We could collect completed information on staging for only 28.6% of participants. Staging was done using the American Joint Committee on Cancer's staging system [21]. In the first step, staging was done clinically using history of dysphagia and other major symptoms and routine laboratory analyses, such as liver function tests, plus (1) abdominal sonography and chest x-ray and/or (2) spiral CT scan of thorax and abdomen [20]. When such information was not available, the tumor stage was classified as ‘unknown’. Otherwise, tumors were staged as following. ESCC was categorized as stage IV when there was evidence of distant metastasis. In other cases, the final staging was dependent on information regarding local progression of tumor, such as data from endoscopic ultrasonography and/or surgery. When such information was not available, tumors were included among those with ‘unknown’ staging. Stage I was assigned when tumor invaded lamina propria or submocusa only. Tumors were classified as stage II when tumor invaded muscularis propria or adventitia without any involvement of lymph node or when the extension of tumor was similar to that of stage I but with involvement of regional lymph nodes. Stage III was assigned when tumor invaded adventitia with involvement of regional lymph nodes or when tumor invaded adjacent structures; many of the latter cases were staged in the first step using information from CT scans.

Cigarette, hookah, nass, and opium users were defined as individuals who had ever used the respective substances at least once per week for a minimum duration of 6 months. Those who had started using any tobacco product or opium within one year before diagnosis of their disease were considered as non-users. We did not include alcohol drinking in our analyses, as few participants were alcohol drinker [18]. Demographic and lifestyle characteristics were based on the information that was recorded at diagnosis.

We categorize the treatments that participants received as: (1) no surgery, chemotherapy, or radiotherapy; (2) only surgery; (3) surgery plus chemotherapy; (4) surgery plus radiotherapy; (5) surgery plus chemoradiotherapy; (6) only chemotherapy; (7) only radiotherapy; and (8) only chemoradiotherapy. As detailed information was not available on the chemotherapy and radiotherapy regimens that were used, individuals who received any regimen (even uncompleted) were categorized as recipient of the respective treatment.

Written informed consent was obtained from all participants. The study was reviewed and approved by the Institutional Review Board of DDRC at Tehran University of Medical Sciences.

### Statistical analysis

Survival probability for all participants combined and by treatment was calculated using the Kaplan-Meier method. Hazard ratios (HR) and corresponding 95% confidence intervals (CI) for the influence of several variables of interest on survival were estimated using the Cox proportional hazards regression models. Results for univariate and two multivariate models are presented. The first multivariate model was adjusted for age (categorical), sex, place of residence, ethnicity, nass chewing, and stage of the disease. The second model was further adjusted for the type of treatment received. As an attempt to identify indicators of receiving potentially curative treatment, which showed a strong association with survival, we examined correlation of several demographic factor and nass chewing with receiving surgery and any curative treatment for ESCC. Correlation coefficients and p-values were derived from multivariate logistic models in which treatments were dependent variables and place of residence, ethnicity, age, sex, nass chewing and stage of ESCC (I/II, III, IV, unknown) were included as independent variables. As place of residence was correlated with ethnicity, we examined interaction between these two variables with regard to receiving treatments using similar logistic models. All statistical analyses were performed using Stata (StataCorp LP, TX, USA; version 11) statistical software. All statistical tests were two-sided.

## Results

**Figure 1:**
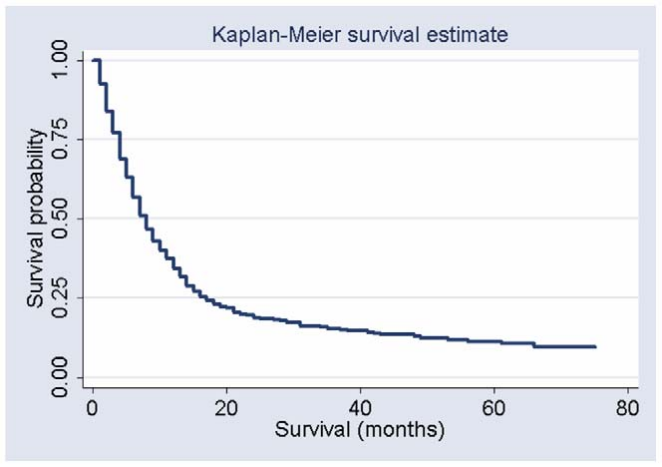
Survival of participants with esophageal squamous cell carcinoma in Golestan Province, Iran.

**Figure 2:**
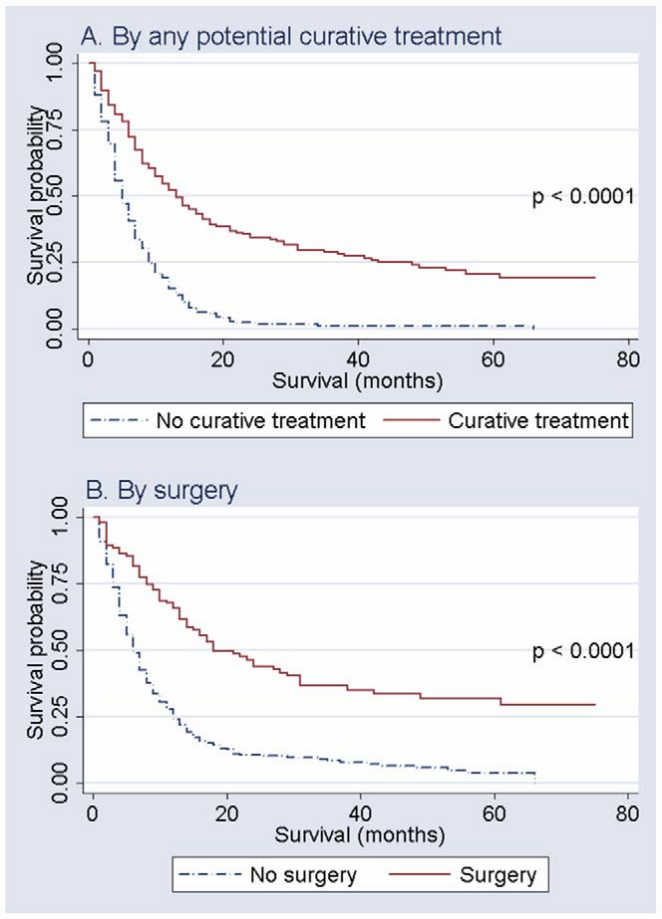
Survival of participants with esophageal squamous cell carcinoma by treatment (p-values come from log-rank tests for equality of survivor functions).

A total of 449 cases of ESCC were eligible for this study. Follow up data were not completed for 23 cases (5.1%) due to change in their living address, so they were excluded from this study and data from 426 individuals were used for current analysis. The overall survival estimation curve is presented in Figure 1. The median survival was 7 months. Five-year survival rate was 3.3%. ESCC cases who received any potentially curative treatment and surgery had longer survival (Figure 2; p-values<0.0001).

The HRs and 95% CIs for patient and tumor characteristics are shown in Table 1. While in the Adjusted Model 1 older age was associated with poorer prognosis, the association was disappeared after further adjustment for treatment. The HR (95% CI) was 1.22 (0.90–1.66) for age 66–75 years and 1.23 (0.85–1.77) for age ≥76 years in the Adjusted Model 2. Men did not have poorer prognosis than women (HR = 1.10; 95% CI 0.87–1.38). Both residing in urban areas (HR = 0.70; 95% CI 0.54–0.90) and being of non-Turkmen ethnic groups (HR = 0.76; 95% CI 0.61–0.96) were associated with better prognosis. With regard to the use of tobacco products and opium, only nass chewing showed significant association with poorer prognosis (HR = 1.57; 95% CI 1.13–2.18). With further adjustment for treatment, the association was still borderline significant (HR = 1.38; 95% CI 0.99–1.92).

**Table 1:** Hazard ratios and 95% confidence intervals for death by patient and tumor characteristics.

		HR (95% CI)		
Characteristics	Number (%)	Non-adjusted Model	Adjusted Model 1^a^	Adjusted Model 2^a^
**Age group (years)**				
<56	90 (21.1)	Reference	Reference	Reference
56–65	116 (27.2)	0.82 (0.60–1.13)	0.86 (0.63–1.19)	0.70 (0.50–0.98)
66–75	144 (33.8)	1.48 (1.11–1.99)	1.65 (1.23–2.22)	1.22 (0.90–1.66)
≥76	76 (17.8)	2.11 (1.52–2.94)	2.05 (1.45–2.89)	1.23 (0.85–1.77)
*P* for trend		<0.001	<0.001	0.01
**Sex**				
Women	215 (50.5)	Reference	Reference	Reference
Men	211 (49.5)	1.17 (0.95–1.44)	1.04 (0.83–1.30)	1.10 (0.87–1.38)
**Place of residence**				
Rural	304 (71.4)	Reference	Reference	Reference
Urban	122 (28.6)	0.78 (0.62–0.99)	0.75 (0.58–0.95)	0.70 (0.54–0.90)
**Ethnicity**				
Turkmen	228 (53.5)	Reference	Reference	Reference
Non-Turkmen	198 (46.5)	0.73 (0.59–0.90)	0.76 (0.61–0.95)	0.76 (0.61–0.96)
**Tobacco and opium use** b				
Cigarette	73 (17.3)	1.05 (0.79–1.38)	0.85 (0.62–1.15)	0.85 (0.62–1.18)
Hookah (water pipe)	33 (7.8)	1.22 (0.84–1.78)	1.32 (0.88–1.98)	1.22 (0.81–1.83)
Nass chewing	50 (11.8)	1.85 (1.36–2.51)	1.57 (1.13–2.18)	1.38 (0.99–1.92)
Opium use	131 (31.0)	1.23 (0.98–1.54)	1.09 (0.86–1.39)	1.06 (0.83–1.35)
**Tumor location**				
Upper One third	38 (8.9)	Reference	Reference	Reference
Middle one third	220 (51.6)	0.83 (0.58–1.21)	0.89 (0.61–1.30)	0.92 (0.63–1.36)
Lower one third	127 (29.8)	0.88 (0.60–1.30)	0.96 (0.65–1.43)	0.94 (0.63–1.40)
Unknown	41 (9.6)	0.62 (0.37–1.04)	0.65 (0.38–1.11)	0.80 (0.47–1.37)
**Staging**				
I/II	43 (10.1)	Reference	Reference	Reference
III	61 (14.3)	1.48 (0.94–2.33)	1.48 (0.93–2.34)	1.09 (0.67–1.76)
IV	18 (4.2)	1.59 (0.87–2.92)	1.93 (1.05–3.56)	1.47 (0.78–2.76)
*P* for trend		<0.001	0.002	0.27
Unknown	304 (71.4)	1.98 (1.36–2.90)	1.83 (1.24–2.70)	1.25 (0.83–1.89)
**Treatment**				
None^c^	208 (48.8)	Reference	Reference	-
Only surgery	43 (10.1)	0.30 (0.21–0.45)	0.32 (0.21–0.49)	-
Surgery+chemotherapy	24 (5.6)	0.19 (0.11–0.33)	0.26 (0.14–0.48)	-
Surgery+radiotherapy	6 (1.4)	0.31 (0.11–0.85)	0.52 (0.19–1.46)	-
Surgery+chemoradiotherapy	29 (6.8)	0.18 (0.10–0.30)	0.18 (0.10–0.31)	-
Only chemotherapy	26 (6.1)	0.79 (0.51–1.22)	1.02 (0.65–1.62)	-
Only radiotherapy	34 (8.0)	0.63 (0.43–0.92)	0.67 (0.46–0.99)	-
Only chemoradiotherapy	56 (13.2)	0.29 (0.20–0.41)	0.30 (0.21–0.44)	-

a Adjusted 1 model included other variables in the list excluding tumor location and treatment. Adjusted model 2 was further adjusted for treatment.

b The reference group was the participants who did not use the respective substance.

c No surgery, chemotherapy or radiotherapy.

Location of tumor was not significantly associated with survival. Only one participant had stage I of the disease, so we combined stages I and II for further analyses. More-advanced stages of ESCC were associated with poorer prognosis in Adjusted Model 1: the HR (95% CI) was 1.48 (0.93–2.34) for stage III, 1.93 (1.05–3.56) for stage IV, and 1.83 (1.24–2.70) for unknown stage compared to stages I and II combined. Except for chemotherapy alone, other therapies were associated with longer survival in the multivariate model (Table 1). The best result was observed with surgery plus chemoradiotherapy (HR = 0.18; 95% CI 0.10–0.31).

Distribution of individuals who received potentially curative treatments was highly varied among categories of place of residence and ethnicity (Table S1). Table 2 shows correlation coefficients (95% CIs) and p-values for several factors and receiving surgery or any curative treatment for ESCC. Place of residence did not show significant correlation with receiving treatment. Being of non-Turkmen ethnic group was correlated with receiving surgery (*P* = 0.006) and any curative treatment (*P* = 0.01). There was no interaction between place of residence and ethnicity with regard to surgery (*P* = 0.92), but the interaction was borderline significant for any curative treatment (*P* = 0.06). Age was strongly correlated with lower rates of receiving treatments. Nass chewer received less curative treatment than non-chewers (*P* = 0.003), but the difference with regard to surgery was not significant.

**Table 2:** Coefficients for correlation of demographic characteristics and nass chewing with receiving surgery or any curative treatment.

	Surgery		Any curative treatment	
	β (95% CI)	*P*	β (95% CI)	*P*
Urban dwellers	0.40 (−0.15–0.96)	0.16	0.31 (−0.17–0.79)	0.21
Non-Turkmens	0.72 (0.21–1.23)	0.006	0.56 (0.13–1.00)	0.01
Interaction of place of residence and ethnicity	0.06 (−1.08–1.20)	0.92	0.94 (−0.04–1.93)	0.06
Age (years)				
56–65	−0.83 (−1.45–−0.21)	0.009	−0.29 (−0.90–0.31)	0.34
66–75	−1.33 (−1.96–−0.70)	<0.001	−0.81 (−1.39–−0.23)	0.006
≥76	−3.44 (−4.94–−1.94)	<0.001	−1.76 (−2.48–−1.04)	<0.001
Men	0.43 (−0.08–0.93)	0.10	0.36 (−0.08–0.79)	0.11
Nass chewers	−0.72 (−1.68–0.24)	0.14	−1.07 (−1.78–−0.36)	0.003

Correlation coefficients and p-values come from multivariate logistic models in which all other variables listed in the table (excluding the interaction) and stage of ESCC (I/II, III, IV, unknown) were included. Reference categories were rural dwellers, Turkmens, age <56 years, women and those who never chewed nass.

## Discussion

Our study showed very poor prognosis of ESCC in Golestan. We found worse prognosis of ESCC with higher age, residing in rural areas, being of Turkmen ethnic group, history of nass chewing, having more advanced disease, and not receiving potentially curative treatments. Turkmens, older patients and nass chewers were less likely to receive curative treatments.

Five-year survival rates of ESCC cases are usually less than 20% [22]. The majority of reports on survival among ESCC cases come from hospital-based studies that investigated treatment modalities and relevant prognostic factors in relation to survival of selected patients undergoing those treatments, such as surgery. There are also several studies that reported survival rates for ESCC cases in the general population [7], [11], [12], [13], [23], [24], [25], [26], [27], [28]. In the majority of these studies, five-year survival rates were reported as approximately 10% [11], [12], [13], [24], [27], [28]. Survival rates in our study were consistent with those from a recent study in northwest of Iran, in which overall survival was reported as 40.5% for one year, 6.5% for three years, and 0.8% for five years [23]. The survival rates in both above studies in Northern Iran and in some other low- to moderate-income countries [29], [30] are generally lower than the rates reported in studies from high-income countries (see above).

Consistent with the literature [27], [8], [25], survival was inversely associated with age in our study. This association remained significant even after adjustment for stage of disease and demographic and lifestyle characteristics. However, this association was strongly attenuated with adjustment for treatment, suggesting that survival among older and younger ESCC cases may not be much different if the same treatment can be administered. Our study showed an inverse correlation between age and receiving surgery or any curative treatment for ESCC. Older patients may be more likely to be considered as not eligible for or refuse more aggressive treatments, particularly surgery. Therefore, they may not receive the treatments that are usually administered to younger patients with a similar stage of disease.

The association of ethnicity/race [31], [25], [32] and place of residence [31] with survival of esophageal cancer cases have been reported in several studies mainly from United States. These associations are not totally explainable by difference in stage at diagnosis. The disparity has been attributed to several factors, including lower socioeconomic status, a later stage at presentation, a higher rate of comorbidities, more difficult access to medical centers, and a lower rate of surgical resection in certain populations. Both place of residence and ethnicity were significant prognostic factors in current study, and ethnicity showed a strong correlation with receiving potentially curative treatments for ESCC, with a potential effect modification from place of residence (but not with regard to surgery). Iran has nationwide health insurance coverage for the inpatient services that are provided in public hospitals. Health insurance for outpatient services is provided to some degree for rural dwellers; for other individuals, it depends on the coverage provided by workplaces for non-short term employees and their family members. On the other hand, although esophagectomy is done in the study area (Eastern Golestan), the hospital volume for providing the surgery and its intensive post-operation care can be limited. Furthermore, no medical canter in Eastern Golestan provides all potentially curative treatments, i.e. surgery, chemotherapy, and radiotherapy, together. Therefore, ESCC patients have to travel to other cities to receive combination therapies. More patients from urban than rural areas may afford frequent travelling, and consequently they may more easily consent to more appropriate and receive more complete treatments, which may take longer durations particularly with radiotherapy and chemotherapy. However, we did not have data on initially proposed treatments by doctors and on completeness of treatment. Potential cultural differences between Turkmens and non-Turkmens in seeking for and/or giving consent to more aggressive treatments, including esophagectomy, may also play a role.

A few studies have reported an association between tobacco smoking and poor prognosis in esophageal cancer [13], [14], [15]. However, such an association was not reported from Northwest of Iran [23]. We found no association between cigarette smoking and survival, but nass chewing was associated with poorer prognosis in our study. Although tobacco chewing has been linked to development of ESCC [33], [34], little information is available on the association between this habit and prognosis of ESCC. We can only speculate on the reasons for this association. Other smoking-related disorders may contribute to earlier death of ESCC cases. In this case, it should also be true for many other cancer types. Tobacco may have a direct effect on tumor progression or may increase mortality from complications of treatment modalities, but we did not observe a similar association with cigarette smoking. Other components of nass may also have adverse influences. On the other hand, nass chewing is much more common in rural areas of Golestan, particularly among Turkmen males [35]. Another explanation could be that nass chewing may be a proxy for another poor prognosis factor that is associated with residing in rural areas and being of Turkmen ethnic group. Further studies are required to investigate whether or not the association between nass and survival in ESCC cases is casual. The use of opium as a traditional treatment for pain, diarrhea, and insomnia in Golestan is fairly common [35]. Although opioids may promote angiogenesis and proliferation of tumor cells in vitro and in vivo [36], [37], we did not find a significant association between opium use and survival of ESCC cases. This is similar to results of a study in Northwest of Iran, which had a small number of opium users though [23].

Similar to the previous literature, more advanced disease was associated with poorer prognosis. On the other hand, patients with advanced cancer at the time of diagnosis may die earlier than those with less-advanced disease due to lead time bias. In our study no screening was planned; patients were referred to Atrak Clinic for clinical diagnosis of ESCC, and in the majority of cases ESCC was diagnosed for the first time. We did not have reliable information regarding the starting time of symptoms to take into account lead time bias in our models. However, because dysphagia as the chief complain of patients happens quiet late in the process of disease and ESCC is a rapidly-progressing cancer, we believe that there was no large variation in the time between the start of symptoms and visiting the clinic, and consequently, major lead time bias was unlikely.

Surgical resection is the gold standard for management of patients with resectable ESCC [13], [38]. However, fewer than half of newly diagnosed cases have totally resectable cancer [39]. The proportion of esophageal cancer cases who present with advanced disease may be higher in some populations, particularly in low/middle income countries [29], [24]. Other therapeutic modalities are usually used when surgical resection is not feasible or not consented. However, combination of chemotherapy and/or radiotherapy with surgery may be associated with more favourable prognosis than surgery alone [38], [40]. In our study, potentially curative treatment was also strongly associated with a more favourable prognosis. An earlier study in Northern Iran reported survival rates among ESCC patients treated with radiotherapy. The rates were 42% for one-year, 11% for three-year, and 8% for five-year survival [41]. Another study reported a much higher five-year survival rate (42%) for the ESCC cases who received combination therapy [42]. However, approximately 45% of cases in that study had stage I or II of the disease. Results of our study did not come from a randomized trial, so they should not be considered as a guideline for treatment of ESCC in the region. Nevertheless, they indicate the importance of availability of relevant facilities and encouraging ESCC cases to undergo potentially curative treatments when indicated.

An association between sex and survival in multivariate models has been reported, with women having longer survival [11], [39], [43], [44]. However, several other studies did not show such a difference [23], [24], [41], [25] or reported different patterns according to treatment modalities [12]. In our study, men and women had little difference in terms of survival.

The main strengths of the current study include the population-based design with a large number of incident cases and high success rate of follow up (completeness of follow up = 94.9%). As one of major limitations of our study, staging data were not available for many participants. We considered the participants with unknown staging as a separate group; the overall prognosis of which was in between of stages III and IV, suggesting that the majority of those participants had stages III or IV. Most of the available staging data in our study were mainly based on the imaging procedures, such as CT scan, that may have limited sensitivity in assessing lymph nodes and identifying metastasis and can lead to misclassification [45], [46]. However, because the majority of cases presented with advanced disease, in many cases imaging procedures were sufficient to detect local or distant extension of tumor. For the above reasons, we do not expect considerable distortion of other associations in the adjusted models by current staging data. Data on socioeconomic status indicators, including education level, was only available for less than half of participants. The other participants had been enrolled in the pilot phase of the Golestan Case-Control Study, in which data on personal education level were not collected. When we considered socioeconomic status for the subgroup for which such information was available, study power was not enough to derive reliable conclusions. Therefore, we were not able to investigate socioeconomic indicators in relation to survival. We did not have data on access of the study participants to treatment and cancer care facilities, as well as on patients' compliance to different treatment modalities. This limits our conclusion on relation of demographic variables to survival.

In summery, prognosis of ESCC in Golestan is very poor. We found several factors that were associated with survival of ESCC cases, among which were stage of disease and history of receiving potentially curative treatments. Easier access to treatment facilities may improve the prognosis of ESCC in Golestan. We observed an association between nass chewing and poorer prognosis, whereas tobacco smoking was not associated with prognosis of ESCC. This association may suggest a possible role for ingestion of tobacco and/or other nass constituents in prognosis of ESCC. As survival of ESCC patients is still poor worldwide, it is also essential to identify and reduce the exposure to preventable risk factors of ESCC.

## Supporting Information

Table S1 Age, stage of ESCC, and treatment distributions by place of residence and ethnicity.
(DOC)

## References

[1] Kamangar F, Dores GM, Anderson WF (2006). Patterns of cancer incidence, mortality, and prevalence across five continents: defining priorities to reduce cancer disparities in different geographic regions of the world.. J Clin Oncol.

[2] Jemal A, Center MM, Desantis C, Ward EM (2010). Global patterns of cancer incidence and mortality rates and trends.. Cancer Epidemiol Biomarkers Prev.

[3] Blot WJ, McLaughlin JK, Fraumeni JF, Schottenfeld D, Fraumeni JF (2006). Esophageal Cancer.. Cancer Epidemiology and Prevention.

[4] Trivers KF, Sabatino SA, Stewart SL (2008). Trends in esophageal cancer incidence by histology, United States, 1998–2003.. Int J Cancer.

[5] Steevens J, Botterweck AA, Dirx MJ, van den Brandt PA, Schouten LJ (2010). Trends in incidence of oesophageal and stomach cancer subtypes in Europe.. Eur J Gastroenterol Hepatol.

[6] Islami F, Kamangar F, Aghcheli K, Fahimi S, Semnani S (2004). Epidemiologic features of upper gastrointestinal tract cancers in Northeastern Iran.. Br J Cancer.

[7] Klint A, Engholm G, Storm HH, Tryggvadottir L, Gislum M (2010). Trends in survival of patients diagnosed with cancer of the digestive organs in the Nordic countries 1964–2003 followed up to the end of 2006.. Acta Oncol.

[8] Wang LS, Chow KC, Chi KH, Liu CC, Li WY (1999). Prognosis of esophageal squamous cell carcinoma: analysis of clinicopathological and biological factors.. Am J Gastroenterol.

[9] Peyre CG, Hagen JA, DeMeester SR, Van Lanschot JJ, Holscher A (2008). Predicting systemic disease in patients with esophageal cancer after esophagectomy: a multinational study on the significance of the number of involved lymph nodes.. Ann Surg.

[10] Lin CS, Chang SC, Wei YH, Chou TY, Wu YC (2009). Prognostic variables in thoracic esophageal squamous cell carcinoma.. Ann Thorac Surg.

[11] Trivers KF, De Roos AJ, Gammon MD, Vaughan TL, Risch HA (2005). Demographic and lifestyle predictors of survival in patients with esophageal or gastric cancers.. Clin Gastroenterol Hepatol.

[12] Steyerberg EW, Neville B, Weeks JC, Earle CC (2007). Referral patterns, treatment choices, and outcomes in locoregional esophageal cancer: a population-based analysis of elderly patients.. J Clin Oncol.

[13] Sundelof M, Lagergren J, Ye W (2008). Patient demographics and lifestyle factors influencing long-term survival of oesophageal cancer and gastric cardia cancer in a nationwide study in Sweden.. Eur J Cancer.

[14] Cescon DW, Bradbury PA, Asomaning K, Hopkins J, Zhai R (2009). p53 Arg72Pro and MDM2 T309G polymorphisms, histology, and esophageal cancer prognosis.. Clin Cancer Res.

[15] Shitara K, Matsuo K, Hatooka S, Ura T, Takahari D (2010). Heavy smoking history interacts with chemoradiotherapy for esophageal cancer prognosis: a retrospective study.. Cancer Sci.

[16] Semnani S, Sadjadi A, Fahimi S, Nouraie M, Naeimi M (2006). Declining incidence of esophageal cancer in the Turkmen Plain, eastern part of the Caspian Littoral of Iran: a retrospective cancer surveillance.. Cancer Detect Prev.

[17] Cook-Mozaffari PJ, Azordegan F, Day NE, Ressicaud A, Sabai C (1979). Oesophageal cancer studies in the Caspian Littoral of Iran: results of a case-control study.. Br J Cancer.

[18] Nasrollahzadeh D, Kamangar F, Aghcheli K, Sotoudeh M, Islami F (2008). Opium, tobacco, and alcohol use in relation to oesophageal squamous cell carcinoma in a high-risk area of Iran.. Br J Cancer.

[19] Abnet CC, Saadatian-Elahi M, Pourshams A, Boffetta P, Feizzadeh A (2004). Reliability and validity of opiate use self-report in a population at high risk for esophageal cancer in Golestan, Iran.. Cancer Epidemiol Biomarkers Prev.

[20] Thompson WM, Halvorsen RA, Foster WL, Williford ME, Postlethwait RW (1983). Computed tomography for staging esophageal and gastroesophageal cancer: reevaluation.. AJR Am J Roentgenol.

[21] American Joint Committee on Cancer (2002). AJCC Cancer Staging Manual.

[22] Jemal A, Siegel R, Xu J, Ward E (2010). Cancer statistics, 2010.. CA Cancer J Clin.

[23] Samadi F, Babaei M, Yazdanbod A, Fallah M, Nouraie M (2007). Survival rate of gastric and esophageal cancers in Ardabil province, North-West of Iran.. Arch Iran Med.

[24] Yeole BB, Kumar AV (2004). Population-based survival from cancers having a poor prognosis in Mumbai (Bombay), India.. Asian Pac J Cancer Prev.

[25] Greenstein AJ, Litle VR, Swanson SJ, Divino CM, Packer S (2008). Racial disparities in esophageal cancer treatment and outcomes.. Ann Surg Oncol.

[26] Stockeld D, Backman L, Fagerberg J, Granstrom L (2007). Esophageal cancer in Stockholm county 1978–1995.. Acta Oncol.

[27] Faivre J, Forman D, Esteve J, Gatta G (1998). Survival of patients with oesophageal and gastric cancers in Europe.. Eur J Cancer.

[28] Baastrup R, Sorensen M, Hansen J, Hansen RD, Wurtzen H (2008). Social inequality and incidence of and survival from cancers of the oesophagus, stomach and pancreas in a population-based study in Denmark, 1994–2003.. Eur J Cancer.

[29] Alidina A, Gaffar A, Hussain F, Islam M, Vaziri I (2004). Survival data and prognostic factors seen in Pakistani patients with esophageal cancer.. Ann Oncol.

[30] Abdullah M, Karim AA, Goh KL (2010). Late presentation of esophageal cancer: observations in a multiracial South-East Asian population.. J Dig Dis.

[31] Jeffreys M, Stevanovic V, Tobias M, Lewis C, Ellison-Loschmann L (2005). Ethnic inequalities in cancer survival in New Zealand: linkage study.. Am J Public Health.

[32] Dubecz A, Sepesi B, Salvador R, Polomsky M, Watson TJ (2010). Surgical resection for locoregional esophageal cancer is underutilized in the United States.. J Am Coll Surg.

[33] Boffetta P, Hecht S, Gray N, Gupta P, Straif K (2008). Smokeless tobacco and cancer.. Lancet Oncol.

[34] Secretan B, Straif K, Baan R, Grosse Y, El Ghissassi F (2009). A review of human carcinogens–Part E: tobacco, areca nut, alcohol, coal smoke, and salted fish.. Lancet Oncol.

[35] Pourshams A, Khademi H, Malekshah AF, Islami F, Nouraei M (2010). Cohort Profile: The Golestan Cohort Study–a prospective study of oesophageal cancer in northern Iran.. Int J Epidemiol.

[36] Gupta K, Kshirsagar S, Chang L, Schwartz R, Law PY (2002). Morphine stimulates angiogenesis by activating proangiogenic and survival-promoting signaling and promotes breast tumor growth.. Cancer Res.

[37] Singleton PA, Lingen MW, Fekete MJ, Garcia JG, Moss J (2006). Methylnaltrexone inhibits opiate and VEGF-induced angiogenesis: role of receptor transactivation.. Microvasc Res.

[38] Kranzfelder M, Buchler P, Lange K, Friess H (2010). Treatment options for squamous cell cancer of the esophagus: a systematic review of the literature.. J Am Coll Surg.

[39] Rouvelas I, Zeng W, Lindblad M, Viklund P, Ye W (2005). Survival after surgery for oesophageal cancer: a population-based study.. Lancet Oncol.

[40] Malthaner R, Wong RK, Spithoff K (2010). Preoperative or postoperative therapy for resectable oesophageal cancer: an updated practice guideline.. Clin Oncol (R Coll Radiol ).

[41] Hajian-Tilaki KO (2001). Factors affecting the survival of patients with oesophageal carcinoma under radiotherapy in the north of Iran.. Br J Cancer.

[42] Salek R, Bezenjani SE, Saedi HS, Ashkiki MH, Hosainzade SM (2009). A geographic area with better outcome of esophageal carcinoma: is there an effect of ethnicity and etiologic factors?. Oncology.

[43] Gaur P, Sepesi B, Hofstetter WL, Correa AM, Bhutani MS (2010). Endoscopic esophageal tumor length: a prognostic factor for patients with esophageal cancer.. Cancer.

[44] Shimada Y, Imamura M, Watanabe G, Uchida S, Harada H (1999). Prognostic factors of oesophageal squamous cell carcinoma from the perspective of molecular biology.. Br J Cancer.

[45] Wallace MB, Nietert PJ, Earle C, Krasna MJ, Hawes RH (2002). An analysis of multiple staging management strategies for carcinoma of the esophagus: computed tomography, endoscopic ultrasound, positron emission tomography, and thoracoscopy/laparoscopy.. Ann Thorac Surg.

[46] van Vliet EP, Heijenbrok-Kal MH, Hunink MG, Kuipers EJ, Siersema PD (2008). Staging investigations for oesophageal cancer: a meta-analysis.. Br J Cancer.

