# Supplementary material for: Prognostic Factors for Esophageal Squamous Cell Carcinoma—A Population-Based Study in Golestan Province, Iran, a High Incidence Area
Source: PLoS One. 2011 Jul 21;6(7):e22152. doi: 10.1371/journal.pone.0022152 (PMC3141005; doi:10.1371/journal.pone.0022152)
Supplement: Table S1 — Age, stage of ESCC, and treatment distributions by place of residence and ethnicity. (DOC) [file pone.0022152.s001.doc]

Supplementary Table 1. Age group, staging, and treatment by the combination of place of residence and ethnicity

|  | Rural, Turkmen | Rural, non-Turkmen | Urban, Turkmen | Urban, non-Turkmen | p-value ^a^ |
| --- | --- | --- | --- | --- | --- |
| **Age (years)** |  |  |  |  |  |
| < 56 | 46 (24.9) | 23 (19.3) | 7 (16.3) | 14 (17.7) |  |
| 56-65 | 53 (28.6) | 36 (30.3) | 10 (23.3) | 17 (21.5) |  |
| 66-75 | 57 (30.8) | 41 (34.5) | 17 (39.5) | 29 (36.7) |  |
| ≥ 76 | 29 (15.7) | 19 (16.0) | 9 (20.9) | 19 (24.1) | 0.55 |
| **Staging** |  |  |  |  |  |
| I/II | 16 (8.6) | 17 (14.3) | 5 (11.6) | 5 (6.3) |  |
| III | 26 (14.1) | 22 (19.5) | 6 (14.0) | 7 (8.9) |  |
| IV | 10 (5.4) | 3 (2.5) | 1 (2.3) | 4 (5.1) |  |
| Unknown | 133 (71.9) | 77 (64.7) | 31 (72.1) | 63 (79.8) | 0.33 |
| **Treatment ^b^** |  |  |  |  |  |
| No | 97 (52.4) | 56 (47.1) | 26 (60.5) | 29 (36.7) |  |
| Yes | 88 (47.6) | 63 (52.9) | 17 (39.5) | 50 (63.3) | 0.04 |
| **Surgery** |  |  |  |  |  |
| No | 150 (81.1) | 85 (71.4) | 34 (79.1) | 55 (69.6) |  |
| Yes | 35 (18.9) | 34 (28.6) | 9 (20.9) | 24 (30.4) | 0.11 |
| **Total** | 185 (100) | 119 (100) | 43 (100) | 79 (100) |  |

^a^ p-values were calculated using chi-squared tests.

^b^ Surgery, chemotherapy, or radiotherapy or any combination of them.
